# Ill, but Still Attractive? The Impact of Mental Illness on Attractiveness Perceptions and Social Judgment

**DOI:** 10.3390/bs14050406

**Published:** 2024-05-13

**Authors:** Nilüfer Aydin, Miriam Clivia Plewe, Luisa Afra Malin Mahr, Janet Kleber

**Affiliations:** Institute of Psychology, University of Klagenfurt, 9020 Klagenfurt am Wörthersee, Austria; miriampl@edu.aau.at (M.C.P.); luisa.mahr@aau.at (L.A.M.M.); janet.kleber@aau.at (J.K.)

**Keywords:** social perceptions, stigma, mental illness, attractiveness, schizophrenia

## Abstract

In line with the “beautiful-is-good” heuristic, numerous studies show that physically attractive individuals are perceived in a more positive light. However, building on previous findings suggesting that the “beauty–good” relationship is bidirectional, the present research investigates how information on a stigmatized attribute impacts attractiveness perceptions and social judgments. Within a controlled experimental design, we present evidence that the mere label of mental illness (i.e., schizophrenia) decreased the positivity of personality evaluations and perceived attractiveness of a male target that had previously been validated to be highly attractive. Consistent with the “good-is-beautiful” heuristic, a mental illness label led to decreased perceptions of attractiveness, which was mediated by the inference of less positive personality characteristics. This finding lends further support for the bidirectional nature of the “beauty–good” relationship and provides a valuable avenue for future research on the multifaceted ways in which the stigma of mental illness affects social perceptions.

## 1. Introduction

The “beautiful-is-good” effect is one of the most enduring and robust findings in (social) psychology. Considerable research over recent decades demonstrates that in almost every context, physically attractive people are highly advantaged, as they generally experience a positive bias in how they are perceived and treated. Even in situations of misbehavior and delinquency, physically attractive people tend to receive more benevolent reactions and judgments than less attractive people [1,2,3,4]. To date, research recognizes the “beautiful-is-good” heuristic as one of the most stable and influential biases in impression formation. However, building on previous findings suggesting that the “beauty–good” relationship is bidirectional in nature (e.g., Gross and Crofton [5]), the present research investigates how information on a stigmatized attribute impacts social judgments and attractiveness perceptions. In line with the “good-is-beautiful” heuristic, we present evidence that an attractive (male) person is evaluated less positively and perceived as less attractive when labeled with a mental illness.

The stigma of mental illness is a ubiquitous and pervasive social phenomenon, leading to severe consequences for those affected by it [6]. Although Western societies have become more open and experienced regarding mental illness, the stigmatization of mental disorders has hardly changed over time, particularly regarding schizophrenia [7,8]. The stigma of mental illness leads to negative stereotyping and emotions, resulting in an increased desire for social distance [9]—social responses that are the opposite of reactions toward physically attractive people. We propose that the label of mental illness biases social judgment regarding personality traits and attractiveness. So far, most of the research in this area has focused on the extent to which conclusions can be drawn from beauty features to health. However, the extent to which an individual’s mental health status can influence personality and attractiveness evaluations remains unclear. The present research fills this gap in the literature. In a well-powered experiment, we tested the hypothesis that describing a target as living with a mental illness (i.e., being diagnosed with depression or schizophrenia) acts as a negative indicator of “goodness”, resulting in less favorable inferences of personality characteristics and decreased attractiveness perceptions.

### 1.1. The “Beautiful-Is-Good” Heuristic

Decades of research have shown that physical attractiveness is a visible characteristic used to draw biased conclusions about various aspects of an individual, influencing subsequent social interactions (e.g., Feingold [10], Langlois et al. [11]). Perceptions of attractiveness are formed by dimensions of both body shape and faces [12,13,14], while some studies suggest that facial attractiveness seems even more important in people’s judgment of overall physical attractiveness (at least in males; [15,16]).

Physically attractive people are typically rated as having favorable personality traits, such as likeability, competence, and trustworthiness, and are commonly perceived as more successful in romantic relationships, job opportunities, and political elections compared to less attractive people [17,18,19]. For example, it was shown that in situations of employment decisions, interviewers’ judgment is significantly biased by the physical appearance of job applicants [18,20]. Moreover, it was found that more attractive candidates are preferred to be supported by voters, even if they have less political experience than less attractive voters [21,22]. The positivity bias toward attractive people seems not to be limited to Western individualistic societies that emphasize uniqueness and distinctiveness; intercultural studies on the attractiveness stereotype do not suggest a significant effect of cultural affiliation, supporting the existence of attractiveness bias also in more collectivistic cultures [23,24,25]. This attributional bias is even observed in situations when physically attractive people display socially undesirable behaviors, such as delinquent [1,3], aggressive [2,4], or antisocial behavior [26]. For example, experimental research by Aydin and colleagues [26] demonstrates that targets displaying excluding behavior experience less aggression, less retaliation, and greater forgiveness by their victims when they are perceived as physically attractive compared to less attractive excluders. This result is partly explained by the fact that good-looking people are perceived as desirable social partners with whom individuals generally want to affiliate. To date, a compelling amount of research supports the notion that physically attractive people are advantaged in social interactions and relationships.

### 1.2. Mental Disorders and Attractiveness

Within the literature that has researched the link between mental illness and attractiveness, most studies focused on attractiveness as the preceding variable. A classic study by O’Grady [27] examined the relationship between sex, physical attractiveness, and the perceived risk of mental illness. The author found that less attractive people were ascribed a significantly higher susceptibility to mental illness. Moreover, it was examined whether attractive people are or are perceived to be mentally healthier than less attractive people [28]. This study was limited to the clinical disorder of depression and compared subjective (self-perceived attractiveness) and objective (actual physical attractiveness) assessments of appearance. The results showed that an increased negative evaluation of subjective appearance, for example, a lower self-assessment of attractiveness, was associated with an increased rate of depressive symptoms. The objective evaluation, the assessment of attractiveness by other people, did not significantly influence depressive symptoms.

Farina and colleagues [29] asked individuals to rate different female targets in terms of their attractiveness based on photos and a live assessment. Unknown to the raters, some of the targets stemmed from a population of psychiatric patients, and others originated from control populations (university employees or shoppers). The results revealed that targets from a psychiatric patient population were rated as significantly less attractive. Considering the association of attractiveness with the adjustment of the targets, as well as the levels of adjustment before disorder onset, the authors concluded that an unattractive appearance may not merely be considered a consequence of mental illness. Rather, the authors reasoned that unattractive (vs. attractive) people are exposed to greater challenges and a less benevolent social environment, rendering low attractiveness a causal risk factor for the development of a mental illness. A replication and extension of this study compared individuals from a psychiatric patient population and three separate control groups of either low, medium, or high socioeconomic status [30]. It was shown that patients with mental illness were rated as less attractive than people of medium and high socioeconomic status. However, the psychiatric patients did not differ significantly from those with low income. Further supporting the reasoning of unattractiveness as a risk factor for mental illness, the study found that attractiveness ratings for psychiatric patients were lower even when based on high school yearbook photos taken long before hospitalization.

Taken together, research in this area has predominantly focused on the impact of (self-) perceived attractiveness on susceptibility to mental illness. Specifically, these findings lend support to the notion that being perceived as unattractive poses a disadvantage and might even be a contributing factor in the development of mental illness. Less is known, however, when it comes to the opposite direction. Specifically, there is a substantial lack of research investigating how individuals with mental illness are perceived in terms of their physical attractiveness and associated social judgments. In fact, based on previous research regarding physical impairments and attractiveness, it can be assumed that individuals without impairments are generally favored over people with disabilities and are thus considered more attractive (e.g., Richardson et al. [31]). However, few studies have explicitly investigated this research question. In a study by Kleck and DeJong [32], using full-body photographs to assess attractiveness, it was found that children with visible impairments were considered significantly less attractive than children without such impairments. Moreover, people reported that they felt less comfortable and were less likely to go out with people with visual impairments, suggesting that a physical impairment negatively affects attractiveness perception [33]. A more complex picture is displayed by Pazhoohi and colleagues [34], who investigated sex differences and the impact of personality traits on perceptions of attractiveness in individuals with physical disabilities of the opposite sex. The authors found that women rated men with physical disabilities as more attractive than men without, while men’s ratings of women’s attractiveness were not affected by physical disability. The authors argue that individual differences in personality and empathy may have a greater impact on the perception of attractiveness than the mechanism in question. Yet, overall, previous research indicates a link between lower attractiveness ratings and physical impairments that change the outward appearance of a target. However, this link might also expand to mental impairments (e.g., mental disorders) that lack externally visible features, but nevertheless carry widespread stigmatization.

### 1.3. Public Stigma of Mental Disorders

The widespread societal phenomenon of public stigma towards mental disorders frequently results in detrimental social outcomes and can be seen as a heavy burden for those labeled with a mental illness [7,9,35]. Individuals with mental illness suffer twice, as, on the one hand, they experience symptoms of their illness and have to cope with the disease itself, and on the other hand, as a symptom of public stigma, they are exposed to prejudice and discrimination by their social environment [8,36]. As a wide range of research shows, individuals may internalize these prejudices and direct them towards themselves, resulting in self-stigmatization [37]. As stated, people with mental illness face various forms of devaluation and discrimination, for example, regarding employment, income, and treatment options in the healthcare system [38].

Reasons for the public stigma against mental illness are of a complex nature, involving aspects of etiological beliefs, stereotypical attitudes and prejudices, and differences in the public perception of different mental disorders [39]. Indeed, public stigma continues to be strongly influenced by the specific type of mental illness that is being considered. Long-term studies show that the public tends to perceive greater social distance from people with schizophrenia compared to other mental disorders like depression [8,40,41]. Hence, people with schizophrenia are still highly severely stigmatized [7,42,43]. Although Western societies have become more accepting and knowledgeable about mental illness over recent decades [43,44], public stigma still remains highly prevalent. Thus, negative stereotypes and prejudicial beliefs perpetuate assumptions that individuals with mental illness are dangerous, criminal, and lack willpower [45]. For example, population surveys show that many people tend to believe that depression is caused by an unhealthy lifestyle and a lack of self-discipline, whereas people with schizophrenia are associated with stereotypes like “dangerous” and “unpredictable” [46]. As a consequence, people tend to maintain a greater social distance from individuals with schizophrenia compared to those with depression [40,41].

Moreover, individuals with mental illness are often held accountable for their condition, leading to the assumption that they are responsible for their own mental state [47]. Wood et al. [48] compared public stigma against schizophrenia, depression, and anxiety disorders in terms of negative stereotypes, patient guilt, and inability to recover. The authors found that schizophrenia was associated with the most negative stereotypes and considered the least likely to recover. Furthermore, individuals with depression were less likely to attribute blame to themselves for their illness, while those with schizophrenia were the most likely to do so, suggesting that depression is subject to less public stigma than schizophrenia. Lay people distinguish between mental disorders and react differently to them based on social distance [49]. Depressive individuals were perceived as less of a risk to the general population than people with schizophrenia. According to the authors, patients with schizophrenia were perceived to be more dangerous than would be justified when compared to acts of violence and aggression by people with alcohol abuse and drug addiction problems. Indeed, research points out that the link between the majority of psychiatric disorders and violent behaviors is non-existent. Rather, attitudes toward individuals with mental illness are, in most cases, rooted in stereotypes and biased perceptions [39,50]. In summary, the public stigma of mental illness is pervasive and remains a pervasive social phenomenon. Research shows that the label of being “mentally ill” acts as a heuristic for dangerousness and uncontrollability, activating negative stereotypes and ultimately leading people to socially distance themselves from this group [8,51].

### 1.4. The “Good-Is-Beautiful” Heuristic

Besides the well-documented “beautiful-is-good” heuristic, research has also found evidence for the reverse—what is perceived as beautiful in the first place is, in part, shaped by what is considered “good” [5]. Specifically, a series of studies have documented how non-physical features can alter perceptions of attractiveness. For example, facial attractiveness evaluation seems susceptible to goal-driven processes based on the desired personality characteristics of potential partners [52,53]. Further, perceptions of high abilities [54], description of a target as being honest [55], and prosocial behavior of a target [56] enhanced attractiveness ratings. In contrast, if participants learned about past transgressions that allowed for inferences of an unmoral character, they rated the target as less attractive [57]. Accordingly, it seems that the quality of personality characteristics or information that allows one to draw implicit inferences about an individual to be “good” influences perceptions of attractiveness.

However, besides the widespread presence of public stigma, only few studies exist that have investigated how mental illness labels might affect perceptions of attractiveness. Using a Japanese sample, a series of studies investigated expectations and evaluations of attractiveness toward groups of targets labeled to deviate from endorsed societal norms, including “mental patients” [58]. When asked to rate several abstract groups of people concerning their attractiveness, the abstract group of “mental patients” received comparatively low ratings. In study 2, participants subsequently received two sets of photos, which they were asked to rate for attractiveness. After rating the first set of photos without additional information, participants were informed that the second set of photos would depict, dependent on the experimental condition, either individuals from a “mental patient”, a “homosexual”, or an “artist” population. Average attractiveness ratings were lower if participants were told the second set of photos depicted “mental patients” compared to when told they were depicting “artists”, but did not differ from the attractiveness ratings of the first set of photos. Further, in two subsequent studies, when individuals were asked to select among a range of pre-tested photos which of the targets could likely be assigned to a group of “mental patients”, participants chose photos of individuals less attractive than the average, reflecting the expectation of “mental patients” to be rather unattractive.

Although these studies provide initial indications that the labeling of a mental illness has an impact on perceived attractiveness, they also carry limitations. Specifically, most of the studies focused on “mental patients” as an abstract group or considered expectations of attractiveness, rather than how attractively individuals with a mental disorder are perceived. Additionally, different sets of photos were used when comparing labeled to unlabeled groups of individuals. Moreover, the studies focused on a broad category of “mental patients” (which, additionally, might implicate hospitalization), rather than using specific diagnostic labels. Finally, the studies leave unclear conclusions as to why individuals with a mental illness might be perceived as less attractive.

### 1.5. The Current Research

Research routinely demonstrates that physically attractive people are clearly socially advantaged in terms of social evaluation [10,11,17]. But is this privilege also valid in situations when a person is labeled as “mentally ill”? Given the widespread stigma toward mental illness and findings on the “good-is-beautiful” heuristic, individuals labeled with a mental illness might be disadvantaged when it comes to perceived attractiveness. While a first set of studies indicates that individuals stemming from a population labeled as mentally ill are expected and judged to be less attractive on average [58], there does not exist any experimental research examining the perceived social judgment and attractiveness of “mentally ill” labeled targets who have been rated beforehand as highly attractive. Moreover, the present research is the first to examine how the label of different mental disorders, schizophrenia vs. depression, affects personality judgments and attractiveness ratings of an attractive male target. This study aims to substantially expand the research on attractiveness and mental illness and thus close an existing research gap.

Based on compelling evidence regarding the “good-is-beautiful” heuristic and mental illness stigma, we argue that people use diagnostic labels of mental illness as a marker to infer less favorable personality characteristics of a target, leading to decreased attractiveness perceptions. Accordingly, we hypothesize that (1) an attractive male target diagnosed with a mental illness will be evaluated as less positive (regarding personality traits) and less attractive compared to an attractive target without such a label. Specifically, we argue that (2) based on recent work on public stigma, an attractive male target diagnosed with schizophrenia will be evaluated most negatively compared to a depression and control group. Moreover, we expected that (3) the inference of less favorable personality characteristics would mediate the effect of a mental illness label on perceived attractiveness.

In our experiment, we focused on a male target to test our research questions. Research has shown that attractiveness is consistently beneficial for men, whereas the evaluation of women seems to be more context-dependent and inconsistent [59]. Therefore, we decided to use a male target to test the effect of the mental illness label, as attractiveness ratings should not be influenced by additional unknown contextual factors.

## 2. Materials and Methods

### 2.1. Participants and Design

Potential participants were contacted via convenience sampling, snowball sampling, a university mailing list, and social media. In total, 450 German-speaking volunteers completed the questionnaire (M age = 30.0; SD = 17.4; age range: 18–76 years; female: n = 225; male: n = 145; non-binary: n = 9) without receiving a reward for participation. The sample consisted of 61% students, trainees, and undergraduates, 32% professionals, and 6% unemployed people. In our sample, 23% indicated to suffer from a mental illness themselves, and 59% had contact with people with a mental illness in their immediate environment.

To examine the effect of the mental illness label, we manipulated a between-subject design for whether the described person was labeled as “mentally healthy” or had a diagnosis of depression or a diagnosis of schizophrenia.

### 2.2. Material and Procedure

First, participants’ demographic data were assessed (e.g., age, gender). We then presented a picture of an attractive man. This picture had been morphed based on images of 33 Caucasian men (mainly students from a Central European university, aged between 21 and 30 years), and this morphed face had been rated as highly attractive in previous research [60]. Below the picture, a short personal description was presented that included the manipulation of the mental illness label:

“This is a photo of a 30-year-old man. He lives with his wife in Munich and has a young son who is three years old. He is happy in his job as an employee at a construction company. He has a good relationship with his work colleagues, with whom he goes bowling once a week after work. The man is mentally healthy (vs. The man has been diagnosed with depression/schizophrenia). In his free time, he likes to play soccer or go to the playground with his son. Reliability and honesty are very important to him”.

Afterward, perceived attractiveness was measured with one item on a 7-point scale (1 = not attractive, 7 = very attractive). The personal evaluation was assessed with five items (i.e., “the man is popular/intelligent/successful/competent/a good husband) on a 5-point Likert scale (1 = completely disagree, 5 = completely agree). The items were combined into a composite scale (Cronbach’s Alpha = 0.777). Finally, we asked whether people had a mental illness themselves (yes/no) and whether they had contact with people with a mental illness (yes/no).

### 2.3. Data Analyses

In the first step, an Analysis of Variance (ANOVA) was conducted to examine the effect of the mental illness label (control group of mentally healthy vs. depression vs. schizophrenia) on personality evaluation and perceived attractiveness. Post hoc tests were analyzed using Bonferroni correction to control for error rate inflation. In a second step, to test whether inferred personality characteristics might play a mediating role between mental illness labels and attractiveness, we employed a mediation analysis.

## 3. Results

### 3.1. Perceived Attractiveness

Perceived attractiveness ratings differed by mental illness label conditions (F(2, 447) = 3.18, *p* = 0.042, η^2^ = 0.014). Perceived attractiveness was highest for the mentally healthy person (M = 4.99, SD = 1.36) and lower for the person with a diagnosis of depression (M = 4.82, SD = 1.35) and schizophrenia (M = 4.61, SD = 1.32). This difference, however, was only significant between the mentally healthy and the schizophrenia conditions (*p* = 0.036). All other post hoc comparisons did not reach significance (*p*s > 0.505).

### 3.2. Personality Evaluation

The participants’ evaluation of the person varied depending on the label of mental health condition (F(2, 447) = 3.33, *p* = 0.037, η^2^ = 0.015). The mentally healthy person was evaluated more favorably (M = 3.85, SD = 0.56) than the person with depression (M = 3.72, SD = 0.52) and schizophrenia (M = 3.69, SD = 0.62); however, only the evaluation of the mentally healthy and the schizophrenia conditions varied significantly (*p* = 0.047) (all other *p*s > 0.162).

### 3.3. Mediation Analysis

We conducted a mediation analysis using PROCESS, Model 4 [61], with 5000 bootstrap samples. A Helmert contrast was used to test the effect of the mental illness label: C1 tested the difference between mentally healthy and the presence of a mental illness (healthy = −0.67, depression = 0.33, schizophrenia = 0.33), whereas C2 tested the difference between a diagnosis of depression and schizophrenia (healthy = 0, depression = −0.50, schizophrenia = 0.50). Mental illness contrasts were entered as the independent variables, personality evaluation as the mediator, and attractiveness perception as the dependent variable.

The presence of a mental illness (C1) predicted the personality evaluation (b = −0.14, SE = 0.06, *p* = 0.012), but there was no effect of the different diagnoses (C2) (b = −0.03, SE = 0.07, *p* = 0.629). Perceived attractiveness was predicted by the personality evaluation (b = 0.78, SE = 0.11, *p* < 0.001). Both contrasts did not predict attractiveness ratings significantly (C1: b = −0.17, SE = 0.13, *p* = 0.182; C2: b = −0.19, SE = 0.15, *p* = 0.197). The bootstrapped confidence interval for the indirect effect of C1 on perceived attractiveness through personality evaluation was significant (b = −0.11, SE = 0.05, 95%-CI [−0.218, −0.022]). There was no indirect effect of the different diagnoses on perceived attractiveness through personality evaluation (b = −0.03, SE = 0.05, 95%-CI [−0.133, 0.078]).

### 3.4. Exploratory Analyses: Participants’ Gender as an Additional Factor

**Figure 1:**
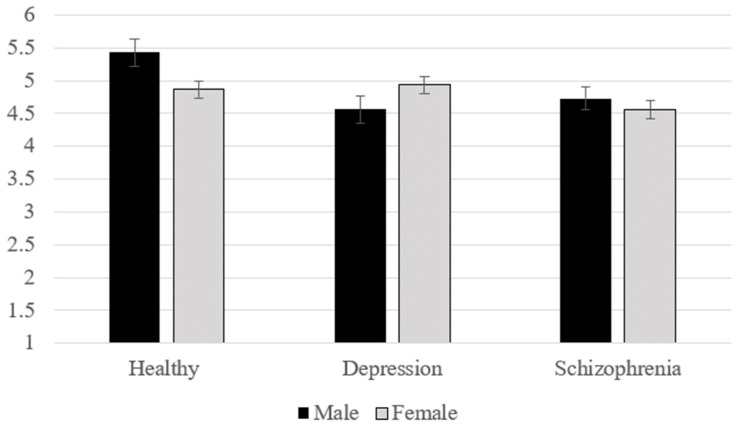
Perceived attractiveness depending on participants’ gender and label condition. Note. Error bars represent standard errors.

Considering the potential relevance of the gender of the participants in the evaluation of the attractiveness of others (e.g., Agthe et al. [62]; Agthe et al. [63]; Nestor et al. [64]), we further investigated in a two-factorial ANOVA the effects of gender (male vs. female) and label conditions (mentally healthy vs. depression vs. schizophrenia) on perceived attractiveness. A significant interaction (F(2, 435) = 3.82, *p* = 0.023, η^2^ = 0.017) revealed that male participants varied in their perceived attractiveness depending on the label conditions (F(2, 435) = 5.11, *p* = 0.006, η^2^ = 0.023). In contrast, female participants did not indicate different attractiveness ratings depending on the label (F(2, 435) = 2.10, *p* = 0.124, η^2^ = 0.010). As depicted in Figure 1, male participants indicated higher attractiveness ratings for the mentally healthy person than for the person with depression (*p* = 0.009) and schizophrenia (*p* = 0.028). Female participants did not differ in their attractiveness ratings (*p*s > 0.156).

Predicting the personality evaluation with participants’ gender and the label condition in a two-factorial ANOVA revealed only a main effect of participants’ gender (F(2, 435) = 10.82, *p* = 0.001, η^2^ = 0.024). Specifically, female participants evaluated the person more favorably (M = 3.83, SD = 0.50) than male participants (M = 3.63, SD = 0.64). Neither the main effect of the condition nor the interaction effect was significant (*p*s > 0.339).

## 4. Discussion

The current study investigated how labels of mental illness affect personality judgments and perceived attractiveness. A plethora of research has shown that attractive individuals are perceived in a more positive light and receive more favorable treatment from others (e.g., Feingold [10], Langlois et al. [11]). We reasoned, however, that individuals with a mental illness might be disadvantaged when it comes to attractiveness perceptions by others. Using a software-manipulated and standardized photo depicting an attractive male (validated for high facial attractiveness in previous research), we found that the mere label of a mental illness significantly flattened attractiveness ratings. Previous research has shown that visible signs of disability (i.e., physical disability) can alter perceived attractiveness (e.g., Kleck and DeJong [32]). Moreover, preliminary findings indicate that individuals labeled to stem from a mental patient population are rated, on average, as less attractive compared to a non-stigmatized control population [58].

However, to the best of our knowledge, our study is the first to show that an identical individual is being judged as less attractive merely due to the description of mental illness, with all other parameters being equal. While we found this effect to be restricted to schizophrenia in our sample (vs. depression), this finding resonates well with the literature, which highlights schizophrenia as one of the most stigmatized mental illnesses, both among the general population and medical professionals (e.g., Angermeyer and Dietrich [50], Valery and Prouteau [51]). For example, in contrast to depression, beliefs about schizophrenia are characterized by greater prognostic pessimism (e.g., Wood et al. [48], Valery and Prouteau [51]), dangerousness, and unpredictability (e.g., Angermeyer and Matschinger [40], Crips et al. [41], Marie and Miles [49]). Of note, the vignette used in our study described the target as socially and professionally integrated and well-functioning. Yet, the literature suggests that the mere presentation of a diagnostic label can initiate the categorization of individuals with mental illness as being part of a distinct outgroup and activate stigma even beyond the presence of psychopathological behavior [65,66,67]. We extend this string of research to perceptions of attractiveness.

While research has routinely demonstrated a “What-is-beautiful-is-good” heuristic, it has also found evidence for the reverse—“What-is-good-is-beautiful” [5]. Specifically, individuals evaluate faces as more attractive when they are perceived to reflect personality characteristics desired in a partner [52]. Likewise, descriptions of individuals that allow to construe valued characteristics heighten perceived attractiveness, while information that allows for negative inferences flattens perceived attractiveness (e.g., Hansson et al. [56], He et al. [57], Paunonen [55]). As such, perceiving someone as attractive might, at least to some extent, also reflect who we consider a desirable interaction and relationship partner. Applied to the area of mental illness, this suggests that individuals use diagnostic labels to infer less valued characteristics that render the target as a non-desirable interaction or relationship partner, which, in turn, decreases attractiveness evaluations. Indeed, previous research has documented social distancing preferences toward targets with mental illness [8,46,51]. Within our mediation analysis, a label of schizophrenia decreased the positivity of the personality evaluations, which, in turn, were associated with reduced attractiveness perceptions.

Given that perceived attractiveness and the subjective evaluation of personality characteristics are necessarily measured rather than experimentally manipulated, the mediation might also work the other way around, with mental illness functioning as a label that more generally renders the individual “less good” in the eyes of the beholder, decreasing attractiveness ratings, which in turn further decreases personality evaluation. In other words, what is considered “good” (being mentally healthy vs. ill) determines what is beautiful in the first step, and what is beautiful determines what is considered good (having favorable personality features) in the second step. Both mediation models (see Appendix A for the reverse mediation model) were significant and, more importantly, can be argued from a theoretical perspective. The association between attractiveness and personality evaluation is most likely one that is bidirectional in nature [5]. Either way, the consequence is that when objectively attractive individuals carry the stigma of mental illness (schizophrenia or depression), they might profit less from a heuristic that equates beauty and goodness. However, both the “beautiful-is-good” and the “good-is-beautiful” accounts suggest that levels of attractiveness and personality ratings should move in tandem, and we are unaware of studies that have documented or could explain the compartmentalization of these two evaluation dimensions.

### 4.1. Exploratory Analyses on Gender Effects

If we included participants’ gender as an additional factor, the pattern changed. Specifically, it showed that only men were affected by diagnostic labels in their attractiveness judgment of a male counterpart, with both labels of schizophrenia and depression resulting in fewer attractiveness ratings compared to a mentally healthy target. Although it is speculative at this point, this gender-specific effect might be rooted in a greater susceptibility of men toward traditional masculinity stereotypes, which often underscore attributes such as strength and emotional control (e.g., Emslie et al. [68], Thompson and Bennett [69]). Previous research has documented a greater tendency among males to erroneously attribute mental illness to personal weakness and that some men experience depression as a challenge to their sense of masculinity [68,70]. At the same time, evidence suggests that men endorse traditional masculinity stereotypes more than women [71]. Thus, the effect on attractiveness ratings being restricted to male participants might reflect a perceived violation of traditional masculinity that is more strongly endorsed by men. However, these results and interpretations should be considered with caution, given their exploratory nature, and that gender differences in attractiveness ratings tend to be unstable [72].

Additionally, this interpretation does not provide clarification as to why diagnostic labels of mental illness affected male assessments of attractiveness but did not modulate personality judgments. Instead, our study only provides evidence for a main effect of gender that female participants hold more favorable evaluations across diagnostic labels, a finding that may reflect a female positivity effect that has been observed for social judgments [73,74].

### 4.2. Limitations and Future Research

The current research provides initial insights into how labels of mental illness can impact social judgments and accompanying perceptions of attractiveness. However, our findings come with some limitations that warrant future investigations. First, within our stimuli, we employed a positive description of the target and used a standardized photo that objectively fulfilled high attractiveness standards (as validated by previous research). While these stimuli set a high benchmark for potential mental illness label effects and thus allowed for a robust test of our hypotheses, these restrictions might have prevented potentially interesting insights. Specifically, extreme cues are weighted more strongly compared to moderate cues in impression evaluation and social judgment [75,76], suggesting that contextual factors like mental illness labels might provide stronger effects once the stimulus material for attractiveness and person description are more moderate or ambiguous (e.g., moderately attractive targets with moderately positive or mixed personal descriptions; cf. Chen et al. [77], Gross and Crofton [5]). Additionally, the implementation of a moderate- or low-attractiveness control group would allow to investigate how mental illness labels moderate effects of high (vs. moderate vs. low) objective attractiveness on perceptions and social judgment by others. Accordingly, to paint a more nuanced and fuller picture of effects, future research would benefit from employing varying levels of attractiveness and personal description stimuli.

Second, it should be noted that our effect sizes are rather small. A current debate in psychological research highlights both the importance and the risks of interpreting small effect sizes [78,79]. Given that we used a very small manipulation that described the target as a socially and professionally integrated and well-functioning person who was either healthy or diagnosed with a mental illness, it is possible that presenting more details about the symptoms of the mental illness or its impact on daily life might have resulted in larger effect sizes.

Third, we implemented a male face as the target stimulus. However, the extent of mental illness stigma can differ with regard to the target’s gender. For example, males with mental illness tend to be judged as more dangerous than females [80], and psychological problems reduce willingness for social interaction more for male than for female targets [81]. Thus, future research could test whether the extent to which mental illness labels reduce attractiveness ratings depends on the target’s gender. Moreover, our exploratory analysis suggested a potential for gender-sensitive effects on the side of the judging person. However, the conclusiveness of these effects is limited insofar as they were only exploratory, and the design excluded the evaluation of same- or cross-gender effects with regard to female targets. Accordingly, future research would benefit from systematically evaluating both target and observer genders. Specifically, our explorative analysis revealed the unexpected finding that the label of mental illness affected male assessments of a male target’s attractivity, but not the person evaluation. This explorative finding warrants replication, and in the case of robustness of the effect, future research is needed to explain how the evaluation of attractiveness and personality judgments might become decoupled under the presence of mental illness labels.

Lastly, future research could tap into domains other than social judgments where attractiveness has been shown to be beneficial (e.g., prosocial behavior [82]) to evaluate whether objectively attractive individuals might profit less when they carry a stigma of mental illness due to reduced attractiveness perceptions.

## 5. Conclusions and Implications

Taking all this together, the current study provides the first and novel evidence that the mere labeling of a person as mentally ill can reduce the positivity of personality judgments and associated attractiveness perceptions. This finding adds further evidence to a bidirectional relationship between inferences of beauty and goodness and suggests that mental illness continues to carry a stigma powerful enough to render individuals to be perceived as “less good”. At the same time, explorative findings insinuate the potential presence of a more complex interplay of mental illness labels and gender. We consider our study as an important starting point for future research to gain a deeper understanding of how mental illness labels can affect attractiveness ratings and downstream consequences. Such insights will help to uncover the multifaceted ways in which the stigma of mental illness affects social perceptions and interpersonal interactions and might ultimately contribute to the development of effective interventional strategies to reduce stigma.

## Data Availability

The data presented in this study are openly available in OSF at https://osf.io/9mt5k/?view_only=85b81a1ea06d44ba9eb8dbbc7b8af1fb, accessed on 3 May 2024.

## Appendix A

Alternative Mediation Model: Mental Illness → Attractiveness → Personality Evaluation

Within an alternative mediation model (PROCESS; Model 4; [61]; 5000 bootstrap samples) we entered the mental illness label as the independent variable, personality evaluation as the dependent variable, and attractiveness rating as the mediator. The presence of a mental illness (C1) predicted attractiveness perceptions significantly (b = −0.28, SE = 0.13, *p* = 0.036), but there was no effect of the different diagnoses (C2) (b = −0.22, SE = 0.17, *p* = 0.168). The personality evaluation was predicted by the attractiveness ratings (b = 0.14, SE = 0.02, *p* < 0.001). Neither contrast reached conventional significance (C1: b = −0.10, SE = 0.05, *p* = 0.054; C2: b < −0.01, SE = 0.06, *p* = 0.976). The bootstrapped confidence interval for the indirect effect of C1 on personality evaluation through attractiveness ratings did not include zero and was, therefore, significant (b = −0.04, SE = 0.02, 95%-CI [−0.086, −0.003]). There was no indirect effect of the different diagnoses on personality evaluation through attractiveness ratings (b = −0.03, SE = 0.02, 95%-CI [−0.080, 0.013]).
